# A Case Report of Infantile Hepatic Hemangioendotheliomas Supplied by the Internal Mammary Artery With Mid-aortic Syndrome, High Cardiac Output, and Right Ventricular Strain

**DOI:** 10.7759/cureus.70594

**Published:** 2024-10-01

**Authors:** Sanjay M Khaladkar, Rohan N Shah, Urvashi Agarwal, Krishnarjun Muralinath

**Affiliations:** 1 Radiodiagnosis, Dr. D. Y. Patil Medical College, Hospital and Research Center, Dr. D. Y. Patil Vidyapeeth (Deemed to be University), Pune, IND

**Keywords:** computed tomography (ct ), high cardiac output, infantile hepatic hemangioendothelioma, mid-aortic syndrome, right internal mammary artery

## Abstract

Hemangiomas affect a wide range of sites, from the brain and musculoskeletal system to visceral organs and skin. Hepatic hemangiomas are benign vascular proliferations of endothelial cells that result in liver lesions. These lesions are congenital if present at birth. However, when they appear after birth during infancy, they are referred to as infantile hepatic hemangiomas (IHHs). These lesions are usually asymptomatic but can occasionally lead to complications such as high cardiac output failure, hypothyroidism, and growth restriction in infants if left untreated. They may also cause mass effects in the liver, making early and timely diagnosis crucial. Despite their histologically benign nature, hepatic hemangiomas can lead to significant morbidity and mortality in affected infants. We describe the case of a male child with tiny, localized, characteristic cutaneous hemangiomas in addition to several hepatic tumors known as infantile hemangiomas (IHs). This case report highlights the varied sites and patterns of involvement, including the chest, trunk, lips, face, scalp, limbs, back, and liver, suggesting multifocal involvement.

## Introduction

Hemangiomas are benign vascular tumors that can affect the skin or internal organs [1]. When these benign proliferations of endothelial cells occur in the liver, they are referred to as infantile hemangiomas (IHs). These tumors are also known as hepatic hemangioendotheliomas and are classified into type 1 and type 2 [2]. Up to 12% of newborns will have an IH by the time they reach one year of age, making them the most prevalent vascular tumor in infancy [3]. IHs can mimic both benign and malignant nonvascular tumors, as well as various forms of vascular abnormalities. Typically, the clinical characteristics aid in making a diagnosis. These tumors go through distinct stages of rapid growth followed by spontaneous involution and cellular proliferation, which can be accelerated by angiogenesis inhibitors [2]. While most lesions are asymptomatic and discovered incidentally during abdominal imaging, some are associated with serious symptoms such as hepatic dysfunction, abdominal compartment syndrome, and high-output cardiac failure [2]. We present a case of IH and focus on its patterns of involvement: focal, multifocal, or diffuse [4].

## Case presentation

**Figure 1:**
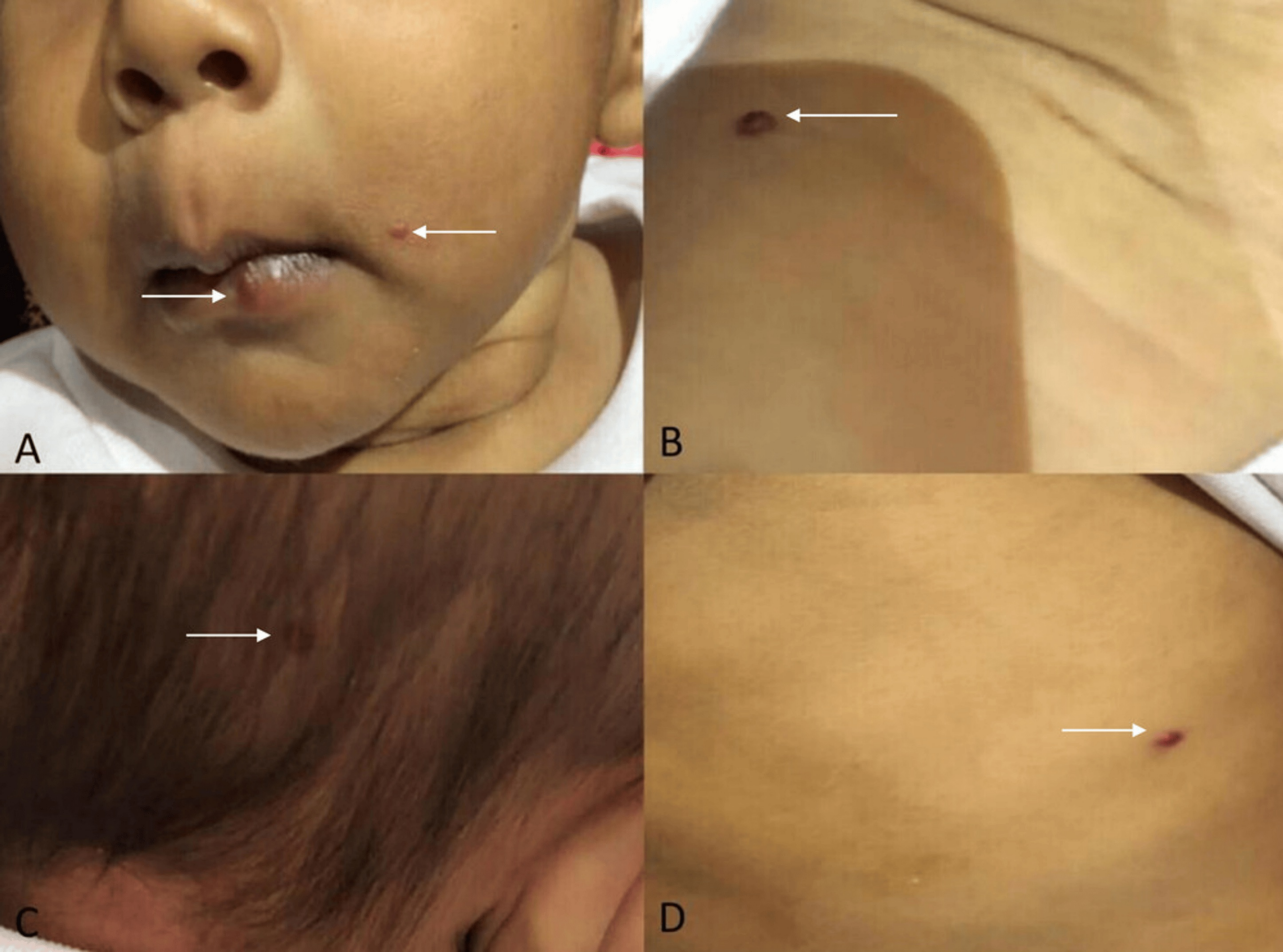
Clinical images showing multiple cutaneous tiny hemangiomas (white arrow) over (A) face and lips, (B) right shoulder, (C) scalp, and (D) trunk

A two-year-old male child was brought to the hospital with complaints of yellowish discoloration of the eyes, vomiting for five days, and red spots on the face, head, and trunk since the fourth day of life. The patient had no significant birth events and was up to date with immunizations. The parents did not report any significant family history. Routine hemograms, liver function tests, and renal function tests were normal. There was no history of previous drug administration. During the physical examination, the patient was vitally stable. Multiple macules and blanching skin lesions were noted diffusely over the body, including the chest (Figure 1D), trunk (Figure 1B), lips (Figure 1A), face (Figure 1A), scalp (Figure 1C), limbs, and back. Respiratory and neurological examinations were normal.

The patient had undergone an ultrasound of the abdomen and pelvis before being brought to the hospital, which showed hepatomegaly with multiple septate cystic lesions with high vascularity. No intrahepatic biliary radicle (IHBR) dilatation or focal mass lesions were noted in the liver. Other abdominal solid organs and visualized bowel loops were reported as normal, with no evidence of ascites.

**Figure 2:**
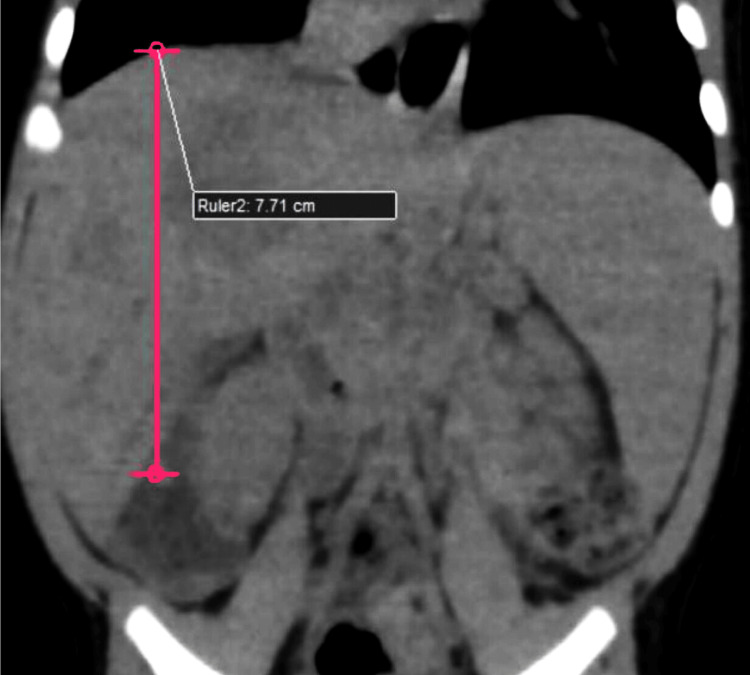
Reformatted coronal section of non-contrast CT image of the abdomen showing hepatomegaly (pink marker) CT: computed tomography

Further imaging included a contrast-enhanced CT scan of the abdomen and pelvis using a Philips 128-slice CT scanner (Philips Healthcare, Best, Netherlands). The contrast administration involved 30 ml of intravenous Iomeron. CT images were acquired in both 5 mm and 1 mm slab thicknesses in axial planes, with parameters of 80 kVp, 1700 mA, a rotation time of 0.4 seconds, and a field of view covering the lower thorax to the upper thighs. The scan was performed in three phases: arterial, portal, and venous, at 40 seconds, 50 seconds, and 60 seconds, respectively. The images were further subjected to 3D and maximum intensity projection (MIP) reconstruction to obtain sagittal and coronal sections. CT imaging revealed hepatomegaly (Figure 2).

**Figure 3:**
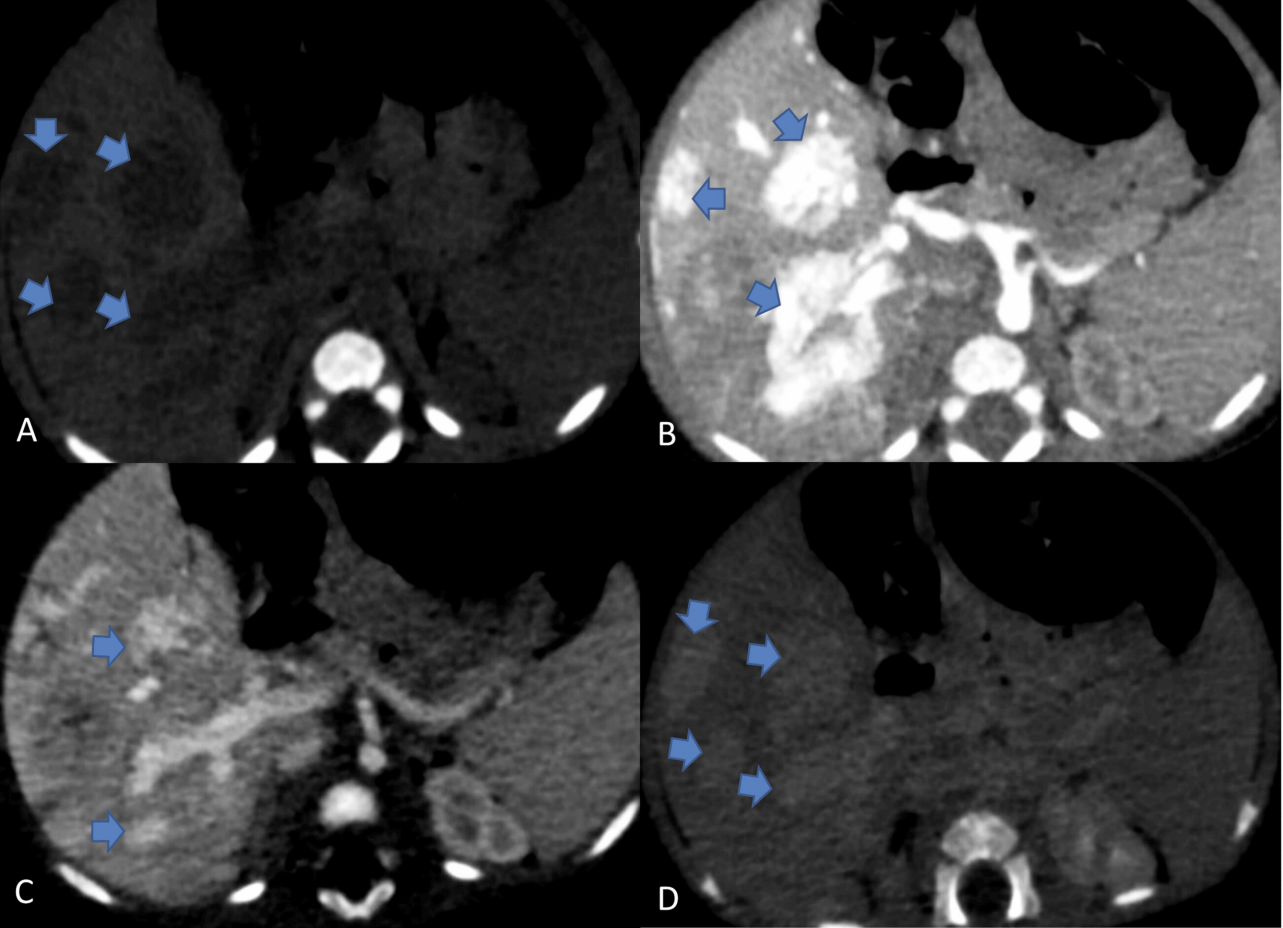
(A to C): axial section contrast-enhanced CT images of the abdomen showing (blue arrows) (A) multiple variable-sized, well-defined round to oval lesions in both lobes of the liver appearing hypodense on the plain study; (B) flash-filling of lesions in the arterial phase; and (C) nodular centripetal enhancement of these lesions in the portal phase. (D) Lesions are slightly hyperdense with respect to normal hepatic parenchyma in delayed phases CT: computed tomography

There was evidence of multiple well-defined, round-to-oval lesions in the liver parenchyma (Figure 3), predominantly in the right lobe. On the arterial phase images, the lesions showed flash filling. In the portal phase, the lesions exhibited nodular centripetal enhancement and became slightly hyperdense compared to the normal hepatic parenchyma in the delayed phases. The largest lesion measured 21 x 25 x 25 mm (CC x AP x TR) in segment VII of the left lobe. Other lesions ranged from approximately 5 to 15 mm in size. No calcification was noted within these lesions, and the intervening liver parenchyma appeared normal in enhancement and attenuation. IHBRs were not dilated.

**Figure 4:**
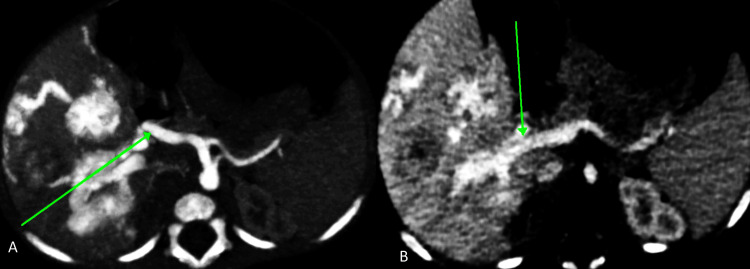
Axial sections of contrast-enhanced CT images showing (A) common hepatic artery (green arrow) and its branches appear dilated and torturous supplying lesions mentioned above; and (B) opacified portal vein (green arrow) and its right and left branches in the arterial phase-suggestive of arterioportal shunting CT: computed tomography

The common hepatic artery, right hepatic artery, and left hepatic artery appeared dilated and tortuous, supplying the hepatic lesions (Figure 4). Arterioportal shunting was indicated by the opacification of the portal vein and its branches in the arterial phase (Figure 4).

**Figure 5:**
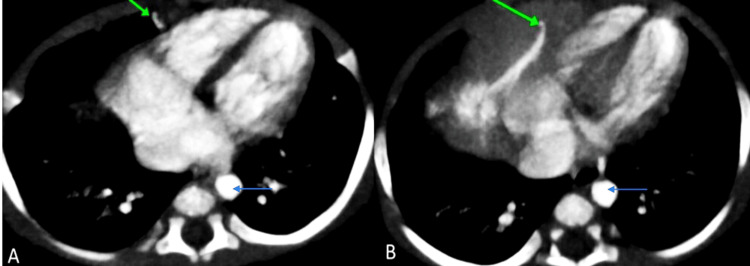
Axial section of contrast-enhanced CT images showing (A) lesion in segment VIII (green arrow) and intense enhancement of IVC (blue arrow) suggestive of right ventricular strain, (B) supplied by right internal mammary artery (green arrow) - likely musculophrenic branch CT: computed tomography; IVC: inferior vena cava

Notably, the lesion in segment VIII was supplied by the right internal mammary artery, likely the musculophrenic branch (Figure 5). Intense opacification of the inferior vena cava (IVC) and hepatic veins indicated right ventricular strain due to high cardiac output (Figure 5).

**Figure 6:**
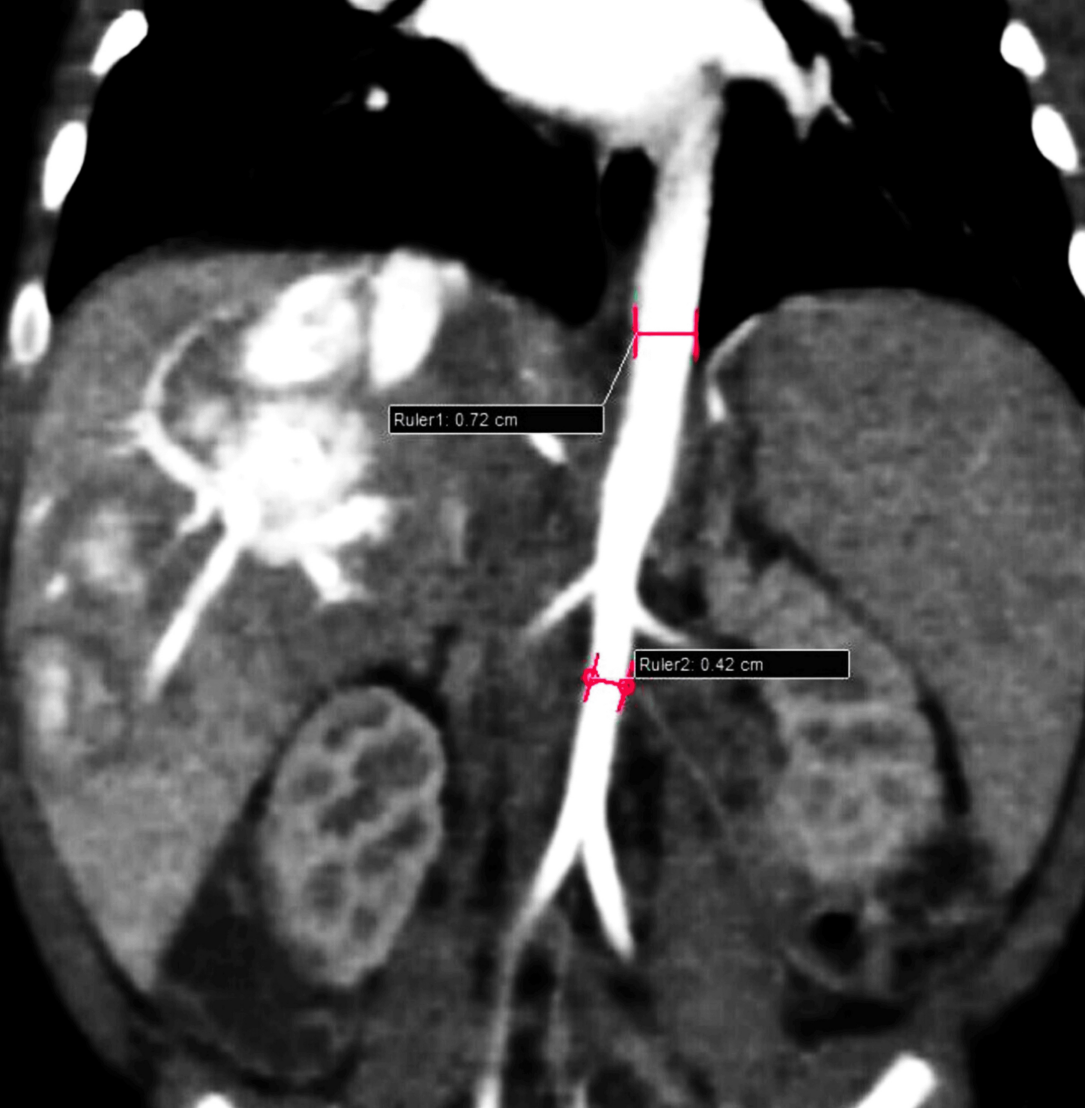
Reformatted coronal section of contrast-enhanced CT image showing narrowing (pink marking) of the aorta distal to celiac trunk - suggestive of mid-aortic syndrome CT: computed tomography

Additionally, narrowing of the mid-aorta was observed, with the proximal aorta measuring 7 mm and the mid-aorta (distal to the celiac trunk) measuring 4 mm, indicative of mid-aortic syndrome (Figure 6).

The gall bladder showed multiple radiodense calculi with CT attenuation values of 180-200 HU, averaging 1-2 mm in size, suggesting cholelithiasis. The clinical and imaging findings are consistent with infantile hemangioendothelioma/hemangiomas. The child was put on steroids to which, he responded and showed improvement.

## Discussion

IH, the most common pediatric malignancy, affects 4-5% of white newborns. It is a benign tumor of endothelial cells that grows rapidly after birth and then slowly involutes during childhood [5]. Reports indicate that 10-25% of babies with cutaneous hemangiomas develop multiple cutaneous IHs [6]. Multiple cutaneous IHs can be a potential indicator of hepatic hemangiomatosis. Patients with cutaneous and hepatic hemangiomas have very different prognoses. While a small proportion of newborns require intensive care, the majority remain asymptomatic and do not need active intervention [6]. The growth and regression patterns of hepatic hemangiomas are similar to those of their more common cutaneous counterparts.

Infantile hepatic hemangiomas (IHHs) can be classified into three types of lesions: diffuse, multifocal, and localized. Focal IHHs are characterized by a single, well-defined, spherical tumor. Areas of heterogeneous enhancement within these lesions may indicate central necrosis, thrombosis, or intralesional hemorrhage. The solid and non-thrombosed regions of the lesion typically show intense, homogeneous enhancement. Most focal lesions are asymptomatic; however, some cutaneous hemangiomas may present with symptoms. Some focal lesions may be associated with minor anemia or thrombocytopenia. Multifocal lesions appear as hypodense lesions with homogeneous enhancement, either uniform or centripetal in pattern. The presence of larger hepatic veins or arteries or aortic tapering (distal to the celiac trunk origin) may suggest arteriovenous shunts. While some multifocal lesions are linked to high-output cardiac failure due to arteriovenous or portovenous shunting, the majority are asymptomatic and discovered incidentally.

Diffuse lesions involve widespread hepatic involvement and nearly complete replacement of the hepatic parenchyma with multiple centripetally enhancing lesions. These lesions tend to have a severe clinical outcome, potentially causing respiratory compromise by compressing the thoracic cavity and IVC. This mass effect can lead to multiorgan system failure and abdominal compartment syndrome, which can be quite severe. Diffuse IHHs can also cause severe hypothyroidism due to an excess of type III iodothyronine deiodinase. This level of hypothyroidism can result in significant mental impairment and heart failure due to weak contractility and low output [5]. Infantile hemangiomas generally show growth regression or involution after the first year of life. However, the proliferation of hepatic masses beyond the first year suggests a malignant tumor rather than benign infantile hemangiomas.

The present case demonstrated typical features of IH, including multifocal skin and hepatic involvement with characteristic imaging features. Medical imaging, being noninvasive, plays a key role in delineating and characterizing hepatic lesions, helping differentiate them from one another. In this case, the hepatic lesion's arterial supply was from the internal mammary artery, which rendered it atypical. Assessing the vascular supply of lesions is important, as most lesions are typically supplied by hepatic arteries [7]. Understanding such variations is crucial for effective diagnosis and appropriate management. Mislabeling IHs as other vascular etiologies can significantly alter management and prognosis. Although a definitive diagnosis is often made histologically, biopsies of hepatic vascular lesions are usually avoided due to the risks of bleeding or death. In such cases, radiologic studies are essential. Radiological imaging is noninvasive and has minimal complications, providing critical information about spatial localization and the exact site of the lesion. The available treatments include interferon alfa-2a, steroids, embolization, and, less commonly, radiation therapy, chemotherapy, surgery, or liver transplantation [2].

## Conclusions

IH is a known but rare entity. If left undiagnosed, it can result in many complications. This report highlights the various complications associated with IH. It also emphasizes the significance of identifying both typical and atypical characteristics, as well as associated complications, of IH, which may initially present nonspecific findings. Hence, early detection and timely management are key to a better prognosis and improved patient care.
